# How to do a second trimester anomaly scan

**DOI:** 10.1007/s00404-022-06569-2

**Published:** 2022-05-11

**Authors:** Natalia Carmen Prodan, Markus Hoopmann, Gertruda Jonaityte, Karl Oliver Kagan

**Affiliations:** Department of Obstetrics and Gynaecology, University of Tuebingen, Calwerstrasse 7, 72076 Tübingen, Germany

**Keywords:** Pregnancy, Fetal anomaly scan, Second trimester, Fetal defects

## Abstract

A systematic evaluation of the fetal anatomy as part of the second trimester ultrasound examination in pregnancy is useful in detecting pregnancy complications, fetal abnormalities, and genetic diseases. We aim to illustrate the basic and detailed second trimester scan, according to current international and national guidelines, as well as to our own every-day practice in the Department for Prenatal Diagnosis at the University of Tübingen, Germany.

Ultrasound examination in pregnancy is one of the main pillars of modern obstetric care. It helps to establish an accurate gestational age and enables a timely detection of pregnancy complications such as fetal structural defects and genetic diseases, multiple gestations, fetal growth restriction, amniotic fluid volume abnormalities, abnormal fetal presentation and abnormal placentation. Timing of the ultrasound examination can be broadly divided into the following categories: a first trimester and a second trimester assessment and, in some settings, a third trimester scan.

The second trimester ultrasound assessment focuses on fetal sonoanatomy to detect fetal structural defects, as well as markers for chromosomal or genetic abnormalities. At present, approximately 50% of all fetal abnormalities are detected prenatally, with detection rates varying widely [1]. For some organ-specific malformations, a structured screening programme has been shown to improve detection rates and outcomes [2]. A Cochrane meta-analysis to assess the effectiveness of second trimester screening in a low-risk population has been initiated [3].

Many scientific societies, among them the ISUOG (International Society of Ultrasound in Obstetrics and Gynaecology) [4], the DEGUM (Deutsche Gesellschaft für Ultraschall in der Medizin) [5] and the AIUM (American Institute of Ultrasound in Medicine) [6], as well as national health authorities, such as the NHS (National Health Service) in the United Kingdom [7], have published guidelines, which define the standard sonoanatomical planes needed for a second trimester fetal examination. In general, national guidelines cover the basic scan requirements. Description of additional views required for a detailed examination is usually provided by scientific societies, which suggest that advanced skills are needed for its performance. This is specifically emphasized in the AIUM guidelines [6].

The ultrasound images below illustrate views, which are required for the completion of a detailed second trimester examination as recommended by ISUOG, AIUM and DEGUM, as well as additional planes and structures that are part of the scanning protocol in the Prenatal Diagnostic Department of the University Clinic for Obstetrics and Gynaecology in Tübingen. We have highlighted the images that are mandatory for the examination according to the ISUOG (*), AIUM (+), DEGUM (#) and NHS-FASP (%) guidelines. We also have marked the additional images that we routinely assess in our clinic as “Tuebingen protocol”. These images are not included in the national recommendations. Still, we believe that they provide valuable information for the pregnancy care.

The enclosed series of images were acquired in ideal conditions in patients with low body mass index, fetus in optimal position, normal amniotic fluid, no fibroids and no uterine malformations. They are also obtained in a series of patients. Therefore, we acknowledge that in every-day clinical practice, one may encounter situations where the acquisition of images of such quality is not possible. Still, every attempt should be made to demonstrate all the relevant structures. If this is not possible, it may be necessary to gently move the fetus with the other hand, to ask the patient to turn to the side, to send the patient for a walk and to empty the bladder or finally to rebook the patient 2 weeks later for another detailed ultrasound examination. Any limitation that affects image quality should be noted in the report (see Fig. 1).

**Fig. 1:**
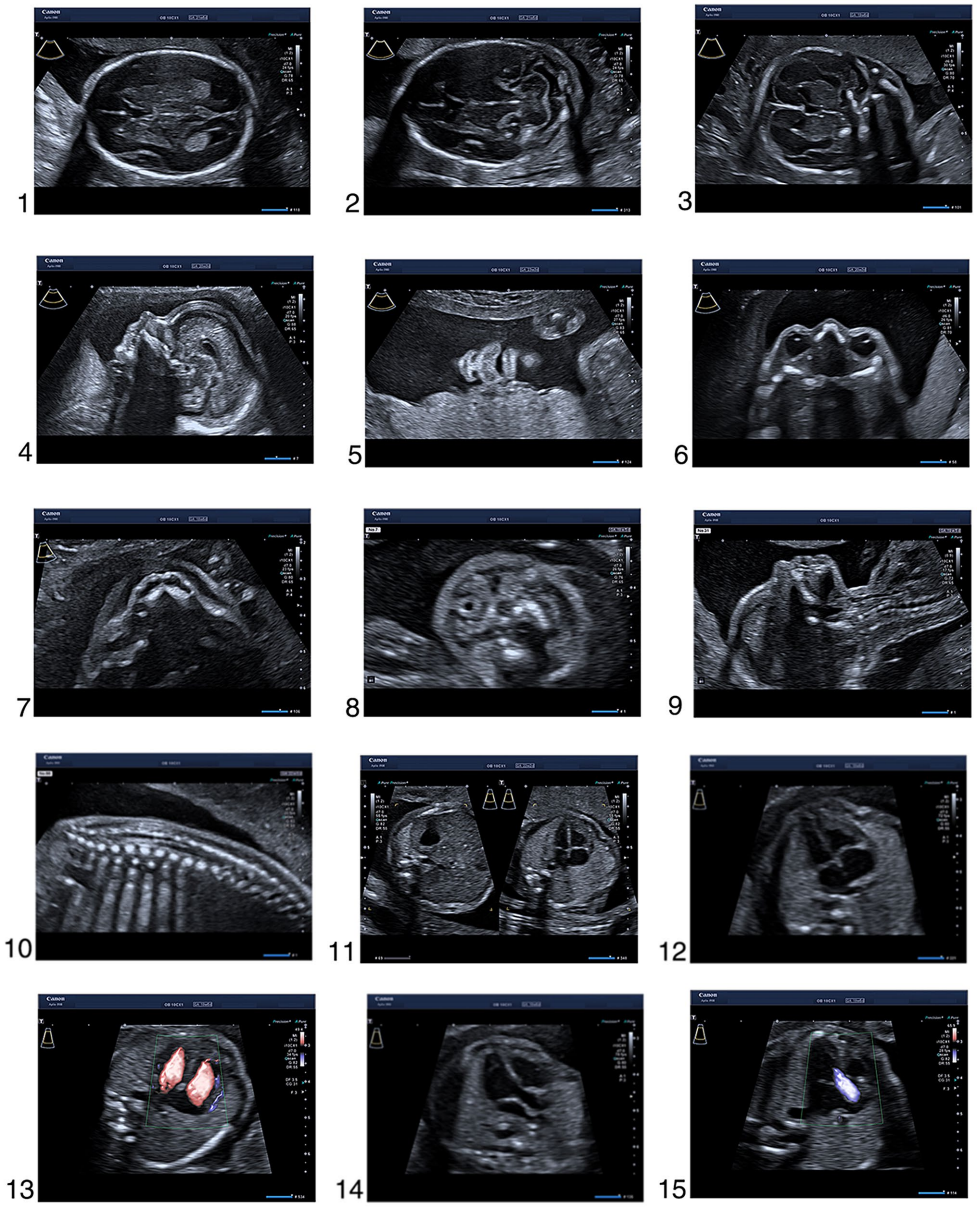

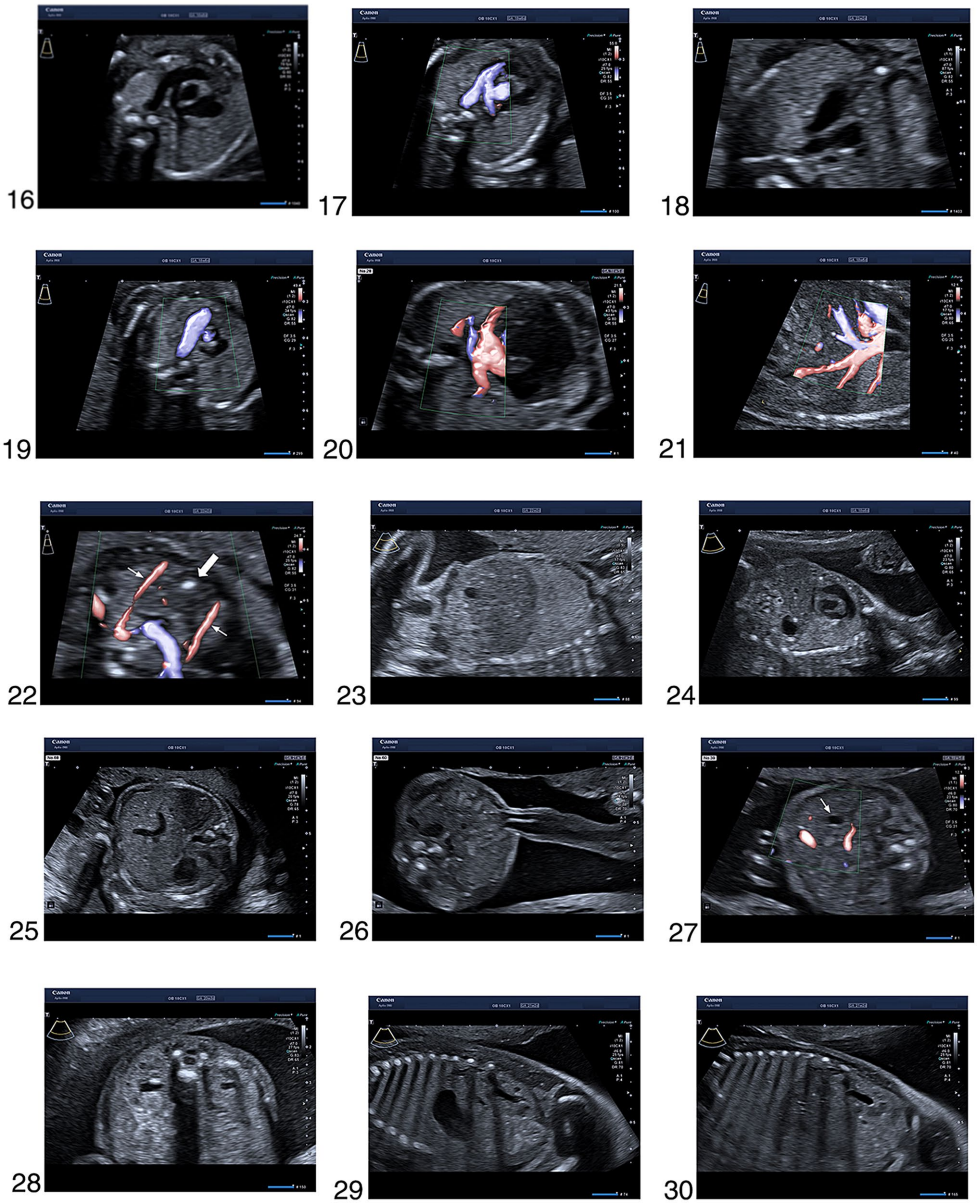

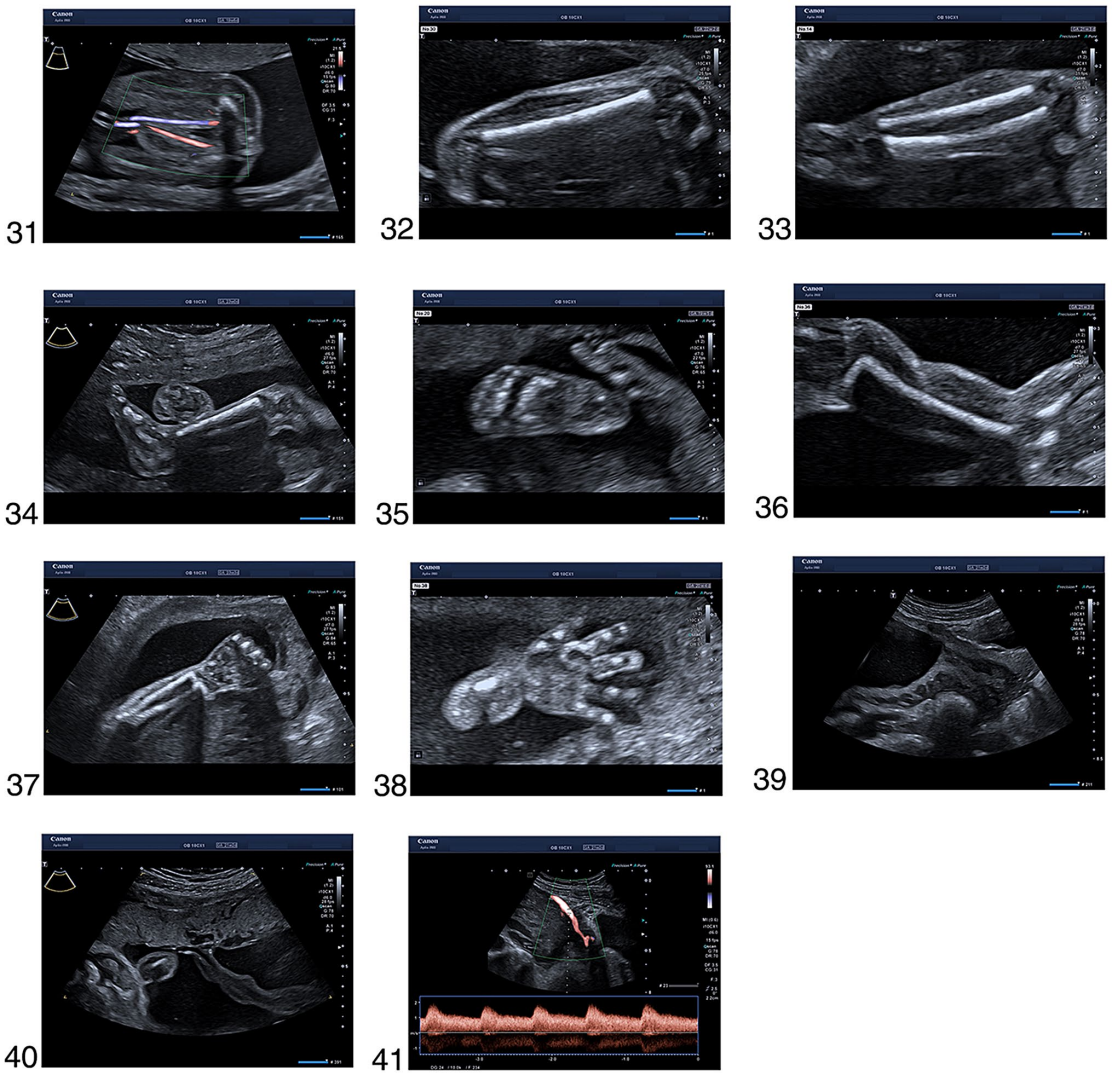
**1**. Transverse image of the head circumference at the level of the biparietal diameter, with midline falx, cavum septi pellucidi, thalami and lateral ventricle (*, + ,#, %), **2.** Posterior fossa of the brain with cerebellum and cisterna magna (*, + ,#, %), **3.** Coronal plane of the fetal head (Tuebingen protocol), ** 4**. Mid-sagittal fetal profile (*, #), ** 5**. Coronal plane of the lips with intact upper lip, mouth, lower lip and nose (*, + ,#, %), **6**. Orbits (*, #) with lenses (Tuebingen protocol), ** 7**. Hard palate (Tuebingen protocol), ** 8**. Transverse plane of the neck with the thyroid gland (arrow) (Tuebingen protocol), ** 9**. Sagittal image of the neck (*, + ,#,%), **10.** Sagittal image of the spine with intact contour of the skin (*, + ,#,%), **11.** Transverse planes of the upper abdomen with the stomach and the thorax with the four chambers of the heart (*, + ,#,%), **12.** Transverse image of the thorax with the apical four chamber view of the heart and the lungs (*, + ,#,%), **13.** Apical four chamber view of the heart in color Doppler (Tuebingen protocol), **14**. Left ventricular outflow tract (*, + ,#,%), **15.** Left ventricular outflow tract in color Doppler (Tuebingen protocol), **16**. Right ventricular outflow tract (*, + ,#,%), **17.** Right ventricular outflow tract in color Doppler (Tuebingen protocol), ** 18**. Three-vessels-view (*, + ,#,%), **19.** Three-vessels-view in color Doppler (Tuebingen protocol) ,** 20**. Pulmonary veins in color Doppler (arrows) (Tuebingen protocol), ** 21**. Inferior vena cava in color Doppler (arrow) (Tuebingen protocol), ** 22**. The thymus (block arrow) between the two internal mammary arteries (arrows) (Tuebingen protocol), ** 23**. Para-sagittal body image with the right diaphragm (#, *), ** 24**. Para-sagittal body image with the left diaphragm (#, *), ** 25**. Transverse image of the abdominal circumference with stomach in normal position (*, + ,#,%), **26.** Insertion of the umbilical cord with intact abdominal wall (*, + ,#), **27.** Gallbladder (arrow) (Tuebingen protocol), ** 28**. Transverse plane of the kidneys showing both renal pelvises (*, + ,#), **29.** Sagittal view of the left kidney (Tuebingen protocol), **30**. Sagittal view of the right kidney (Tuebingen protocol), ** 31**. Bladder with both umbilical arteries (*, + ,#), **32.** Femur (*, + ,#,%), **33.** Frontal view of the shin with tibia and peroneus bone (#), ** 34**. Leg with tibia and correct angle of the talocrural joint (*, + ,#), **35.** Foot (*, + ,#), **36.** Shoulder and humerus (*, + ,#), **37.** Forearm with radius and ulna (#), **38**. Hand (*, + ,#), **39.** Transabdominal view of the cervix (Tuebingen protocol), **40**. Placental insertion of the umbilical cord ( +), **41.** Uterine artery (Tuebingen protocol). *Standard image according to the ISUOG Guidelines. + :Standard image according to the basic fetal examination of AIUM Guidelines. ^#^Standard image according to the DEGUM Guidelines. %Standard image according to the NHS-FASP. Tuebingen protocol: Standard image according to local protocol at the prenatal medicine department at the University Hospital of Tuebingen
